# Site-Directed Alkylation Detected by In-Gel Fluorescence (SDAF) to Determine the Topology Map and Probe the Solvent Accessibility of Membrane Proteins

**DOI:** 10.1038/s41598-019-49292-w

**Published:** 2019-09-11

**Authors:** Yu-Hung Lin, Sung-Yao Lin, Guan-Syun Li, Shao-En Weng, Shu-Ling Tzeng, Yu-Hsuan Hsiao, Nien-Jen Hu

**Affiliations:** 1 Graduate Institute of Biochemistry, National Chung Hsing University, 145 Xinda Rd., South Dist., Taichung City, 402 Taiwan, R.O.C.; 2 Institute of Medicine, Chung Shan Medical University, No.110, Sec. 1, Jianguo N. Rd., Taichung City, 40201 Taiwan, R.O.C.; 3 Rong Hsing Research Center for Translational Medicine, National Chung Hsing University, 145 Xinda Rd., South Dist., Taichung City, 402 Taiwan, R.O.C.; 4 Ph.D. Program in Transnational Medicine, National Chung Hsing University, 145 Xinda Rd., South Dist., Taichung City, 402 Taiwan, R.O.C.

**Keywords:** Structure determination, Transporters

## Abstract

The topology of helix-bundle membrane proteins provides low-resolution structural information with regard to the number and orientation of membrane-spanning helices, as well as the sidedness of intra/extra-cellular domains. In the past decades, several strategies have been developed to experimentally determine the topology of membrane proteins. However, generally, these methods are labour-intensive, time-consuming and difficult to implement for quantitative analysis. Here, we report a novel approach, site-directed alkylation detected by in-gel fluorescence (SDAF), which monitors the fluorescent band shift caused by alkylation of the EGFP-fused target membrane protein bearing one single introduced cysteine. In-gel fluorescence provides a unique readout of target membrane proteins with EGFP fusion from non-purified samples, revealing a distinct 5 kDa shift on SDS-PAGE gel due to conjugation with mPEG-MAL-5K. Using the structurally characterised bile acid transporter ASBT_NM_ as an example, we demonstrate that SDAF generates a topology map consistent with the crystal structure. The efficiency of mPEG-MAL-5K modification at each introduced cysteine can easily be quantified and analysed, providing a useful tool for probing the solvent accessibility at a specific position of the target membrane protein.

## Introduction

The topology of helix-bundle membrane proteins summarizes two-dimensional information of the fold of the polypeptide chain across the cell membrane, i.e. the number, length and orientation of transmembrane segments. With increasing numbers of whole-genome sequences becoming available, *in silico* topology prediction, based on the hydrophobicity analysis of amino acid sequence^1^ and the “positive-in” law^2^, is frequently applied to obtain a topological map of an integral membrane protein. While hydropathy plots illustrate the arrangement of transmembrane segments and thus provide a valuable guide for functional and structural studies, they constitute predictions and, therefore, are often erroneous and require experimental data for validation.

In the past decades, a number of methodologies have been developed for experimental validation of predicted topology maps (for detailed overviews see, for example^3,4^). A common strategy is to construct a chimera fusion with reporter enzymes attached to the terminus of C-terminal truncated target membrane proteins, such as alkaline phosphatase PhoA^5^, β-galactosidase LacZ^6^, and β-lactamase bla^7^. These enzymes show distinct activity in different subcellular compartments. Therefore, the enzyme activity in response to their cellular localization serves as a topological reporter. A similar concept was applied to probe protein glycosylation in eukaryotic cells. Because glycosylation takes place in the lumen of the endoplasmic reticulum (ER), only the glycosylation tag introduced in the luminal loop of ER membrane proteins can be glycosylated, probed by electrophoresis showing molecular weight increase of oligosaccharides attachment^8,9^. Other strategies have been developed on the basis of specific interactions between the probe and target site that only occur in a solvent-exposed compartment. From the viewpoint of experiment design, proteolysis and epitope tags are used for proteolytic enzymes and monoclonal antibody recognition, respectively^10,11^. Because the probing enzymes and antibodies cannot cross the membrane, the distinct pattern obtained in such an analysis will show a lack of interactions between probe and target if the target loop is located intracellularly. Although the methods have successfully demonstrated their capability in topology determination, they share a common problem: it remains unclear whether the inserted tags, either enzymes or sites for glycosylation, proteolysis and immunogenic recognition would disturb the folding and expression of membrane proteins that are subjected to these analyses.

Substituted cysteine accessibility method (SCAM) originally developed by Karlin and co-workers is a versatile method using the covalent interaction between sulfhydryl-specific reagents and cysteines for functional, structural and dynamical studies of membrane proteins^12,13^. Because cysteine is non-bulky in size and relatively hydrophobic, cysteine substitution in most positions of a membrane protein is probably tolerable^4^. SCAM was then applied to define transmembrane orientation by Bagdanov and co-workers, denoted by SCAM^TM^, using membrane-impermeable sulfhydryl-reactive reagents to probe the membrane-embedded and loop regions of membrane proteins in whole cells, right-side-out vesicles, inside-out vesicles, reconstituted proteoliposomes and intact organelles^3,14^. Based on the same concept, Kaback and co-workers developed the site-directed alkylation (SDA) strategies, by labelling the introduced cysteine residues of LacY with sulfhydryl-reactive fluorophores and isotopes, and demonstrated the substrate-induced conformational change responsible for shifting the equilibrium toward the outward-facing state^15,16^. In spite of the effectiveness of the aforementioned methods, detection of the specific modification on target membrane proteins requires labels that afford unique signals for detection, for example by means of chemiluminescence, autoradiography or fluorescence, etc. In most cases, the target membrane proteins have to be purified after labelling.

**Figure 1:**
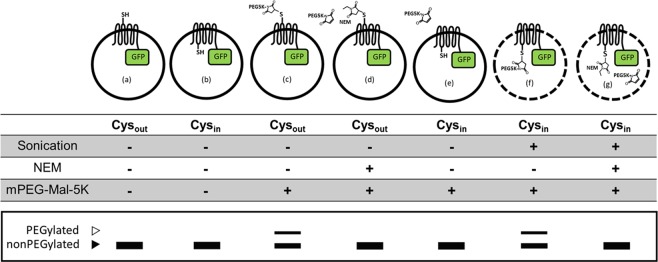
Schematic representation of expected SDAF results indicating the membrane localization of substituted cysteines. The solid and dashed circles represent the intact and permeabilized inner membranes of *E*. *coli* cells, respectively. (**a**,**b**) Non-PEGylated EGFP fusion membrane proteins, bearing one substituted cysteine in the intracellular or extracellular loop, reveal one single fluorescent band using in-gel fluorescence. (**c**) Cysteine residues exposed to the periplasm are accessible to mPEG-MAL-5K because the outer membrane is porous and susceptible to mPEG-MAL-5K permeation. Therefore, PEGylated target membrane proteins reveal a protein band shift. (**d**) Externally facing cysteine residues are blocked by NEM before alkylation with mPEG-MAL-5K and thus the non-PEGylated band is observed. (**e**) Cysteine residues facing the cytoplasm are not PEGylated because the inner membrane is not permeable with respect to mPEG-MAL-5K. (**f**) The cysteine residues facing the intracellular compartments are exposed to mPEG-MAL-5K after membrane permeabilization using sonication. (**g**) NEM treatment after sonication blocks the cysteines to mPEG-MAL-5K and thus the PEGylated band cannot be observed. Structures were generated with the editor cDraw (http://www.structuralchemistry.org/pcsb/cdraw.php).

Here, we present a novel SDA method, termed SDAF, for the determination of transmembrane topology and study of conformational dynamics of membrane proteins with a C-terminal EGFP fusion allowing specific detection of cysteine labelling without any further imaging treatment and protein purification. The sulfhydryl-reactive reagent, methoxypolyethylene glycol maleimide 5 K (mPEG-MAL-5K), covalently attaches to solvent accessible cysteine residues on the surface of intact cells, resulting in a 5 kDa band shift observable by in-gel fluorescence. As illustrated in Fig. 1, being membrane impermeable, mPEG-MAL-5K can only react with cysteine residues in the extracellular loops when performing whole cell PEGylation; it cannot gain the access to cysteine residues in the intracellular loops without disrupting the cell membrane. Application of the SDAF methodology is demonstrated by mapping the topology of the apical Na^+^/bile acid transporter homolog from *Neisseria meningitidis* (ASBT_NM_), the results of which were in agreement with the published crystal structure^17^. Moreover, the degree of PEGylation at given position can be evaluated densitometrically by the ratio of the fluorescence emission of shifted and non-shifted bands. For the example presented, the PEGylation levels of each selected cysteine substitution were in excellent agreement with the solvent accessibility of those residues in ASBT_NM_. In summary, SDAF provides an efficient and direct method for topological mapping and characterization of the conformational variations of membrane proteins in native membrane environment.

## Results

### mPEG-MAL-5K gains access to the cysteine residue on the intracellular side after cell membrane disruption by sonication

**Figure 2:**
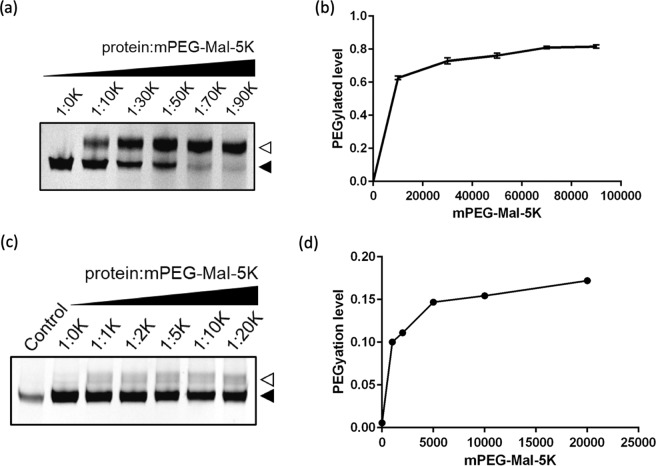
PEGylation efficiencies of ASBT_NM_ cysteine replacements introduced on the extracellular (A275C) and intracellular sides (D61C) at different protein to mPEG-MAL-5K molar ratios. (**a**,**b**) PEGylation of cfASBT_NM_ A275C-EGFP fusion as monitored by in-gel fluorescence using *E*. *coli* whole cells overexpressing cfASBT_NM_ A275C-EGFP treated with mPEG-MAL-5K. Lane 1: cfASBT_NM_ A275C-EGFP without mPEG-MAL-5K treatment. Lanes 2–6: cfASBT_NM_ A275C-EGFP incubated with indicated protein to mPEG-MAL-5K molar ratios. The PEGylated levels of cfASBT_NM_ A275C-EGFP were calculated according to Eq. (1), and plotted against different protein to mPEG-MAL-5K molar ratios. (**c**,**d**) PEGylation of cfASBT_NM_ D61C-EGFP fusion as monitored by in-gel fluorescence using permeabilized membranes treated with mPEG-MAL-5K. Lane 1: *E*. *coli* whole cells overexpressing cfASBT_NM_ D61C-EGFP treated with mPEG-MAL-5K at 1:70 K protein to mPEG-MAL-5K molar ratio. Lane 2: Permeabilized membranes containing cfASBT_NM_ D61C-EGFP without mPEG-MAL-5K treatment. Lanes 3–7: Permeabilized membranes containing cfASBT_NM_ D61C-EGFP treated with indicated protein to mPEG-MAL-5K molar ratios. The PEGylated levels were calculated and plotted as mentioned above. Error bars represent the standard deviation of PEGylation efficiency calculated from three independent experiments. Empty and filled triangles indicate the position of PEGylated and non-PEGylated protein bands, respectively.

Because overexpressed ASBT_NM_ is localized at the inner membrane of *E*. *coli* cells, we first characterize whether mPEG-MAL-5K can permeate through the outer membrane. The outer membrane of *E*. *coli* is porous allowing passage of small molecules with the range of 500–700 Da^18^. Nevertheless, it has been shown that mPEG-MAL-5K can label a solvent accessible cysteine residues of an inner membrane protein on intact *E*. *coli* cell membrane^19^. To validate the hypothesis, we constructed a cysteine-free pWaldo-cfASBT_NM_-EGFP-His_8_ (see Methods) and introduced a single cysteine mutation on the extracellular loop for whole cell PEGylation assay. We initially substituted cfASBT_NM_ A275 (Supplementary Fig. S1a), which is located at the C-terminus of TM9b exposed to the periplasm, with cysteine. The side chain of residue 275 is at a very solvent accessible position and the cysteine sulfhydryl group is situated at the entrance of the outward-facing vestibule for substrate uptake. The PEGylation results showed that cfASBT_NM_ A275C was chemically modified with mPEG-MAL-5K as the fluorescent protein band shifted approximately 5 kDa (Figs 1c, 2a and Supplementary Fig. S1b), indicating mPEG-MAL-5K can permeate the outer membrane of *E*. *coli*. The labelling efficiency, as exemplified in Supplementary Fig. S2 and calculated from Eq. (1), was dose-dependent and reached a saturation (approximately 80%) at a molar ratio of 1:70,000 (ASBT_NM_: mPEG-MAL-5K; Fig. 2b and Supplementary Fig. S1b). As the overexpressed target protein can be quantified using whole cell fluorescence counts, it is straightforward to perform the assay with a constant protein to reagent molar ratio.

It is noted that a band below the fusion protein standing for the size of free EGFP was observed, probably due to non-specific proteolysis during cell lysis in SDS-PAGE sample preparation using *E*. *coli* whole cells. EGFP contains two native cysteine residues, C48 and C70. In a properly folded EGFP molecule, these two cysteines are at the reduced form because they are too distant to form disulphide bond (24 Å). C70 is deeply buried in the β-barrel and C48 is relatively more accessible for sulfhydryl reagents^20^. At the molar ratio of 1:90,000, a faint band shift above the free EGFP is becoming visible (Supplementary Fig. S1b), indicating that EGFP can be labelled with mPEG-MAL-5K in the presence of extremely excessive sulfhydryl reagent. Consequently, we chose the molar ratio of 1:70,000 to perform the subsequent whole cell PEGylation assays.

In order to probe the intracellular loops, we introduced a cysteine at D61 located in the intracellular loop connecting helices TM2 and 3 (Supplementary Fig. S3a). D61C showed no band shifting after PEGylation using intact cells (Figs 1e, 2c and Supplementary Fig. S3b, Control), suggesting mPEG-MAL-5K cannot permeate through the inner membrane. However, the same experiment using disrupted cells revealed a significant band shift with 5 kDa (Figs 1f, 2c and Supplementary Fig. S3b). The labelling efficiency using disrupted membranes also demonstrated a dose-dependent increase and reached a saturation at a molar ratio of 1:5,000 (ASBT_NM_: mPEG-MAL-5K) (Fig. 2d), which was much lower than the ratio in the whole cell PEGylation experiment. This is probably because, in whole cell PEGylation experiments, mPEG-MAL-5K molecules had to penetrate the outer membrane and peptidoglycan to contact the external cysteines; however, in disrupted cells, the physical barriers were destroyed by sonication, making the internal cysteines more accessible to mPEG-MAL-5K. The maximum PEGylation efficiency of cfASBT_NM_ D61C reached a saturation level of 18% at molar ratio of 1:20,000 (Fig. 2d and Supplementary Fig. S2), which was lower than the PEGylation efficiency of cfASBT_NM_ A275C, probably due to the steric hindrance of the head group of lipids, protecting the intracellular cysteine residues from labelling by mPEG-MAL-5K.

### Examining the mPEG-MAL-5K accessibility of the two native cysteine residues in EGFP

In the pWaldo-cfASBT_NM_-EGFP-His_8_ construct, EGFP was fused to the C-terminus of ASBT_NM_ which has a C_in_ topology. Therefore, EGFP is localized on the cytoplasmic side of the *E*. *coli* cell membrane and retains its proper folding for fluorophore formation. For the whole cell PEGylation assay, non-specific modification of mPEG-MAL-5K on C48 and C70 of EGFP is minimized (protein:mPEG-MAL-5K molar ratio = 1:70,000) as the inner membrane of intact cells is impermeable to mPEG-MAL-5K as demonstrated above (Supplementary Fig. S1b). Nevertheless, one cannot rule out the possibility of mPEG-MAL-5K labelling at the two native cysteines of EGFP after sonication disruption. We thus performed a control PEGylation experiment using the sonicator-disrupted membrane fraction containing cfASBT_NM_-EGFP. It revealed a negligible PEGylated band (less than 2% PEGylation efficiency) even at a molar ratio of 1:10,000 (protein:mPEG-MAL-5K; Supplementary Fig. S4), suggesting the two native cysteine residues in folded EGFP are rather inaccessible to mPEG-MAL-5K. In order to minimize the basal PEGylation on EGFP, we therefore utilized a molar ratio of 1:5,000 (protein:mPEG-MAL-5K) in subsequent experiments probing the cysteine replacements in the intracellular loops of cfASBT_NM_, because it almost reached the saturation level of PEGylation (15%) observed with samples of permeabilized membranes containing cfASBT_NM_ D61C-EGFP (Fig. 2d). At this molar ratio, the background PEGylation contributed by the two native cysteine residues of EGFP was less than 1% (Supplementary Fig. S4b).

### Topological mapping of ASBT_NM_ using SDAF

**Figure 3:**
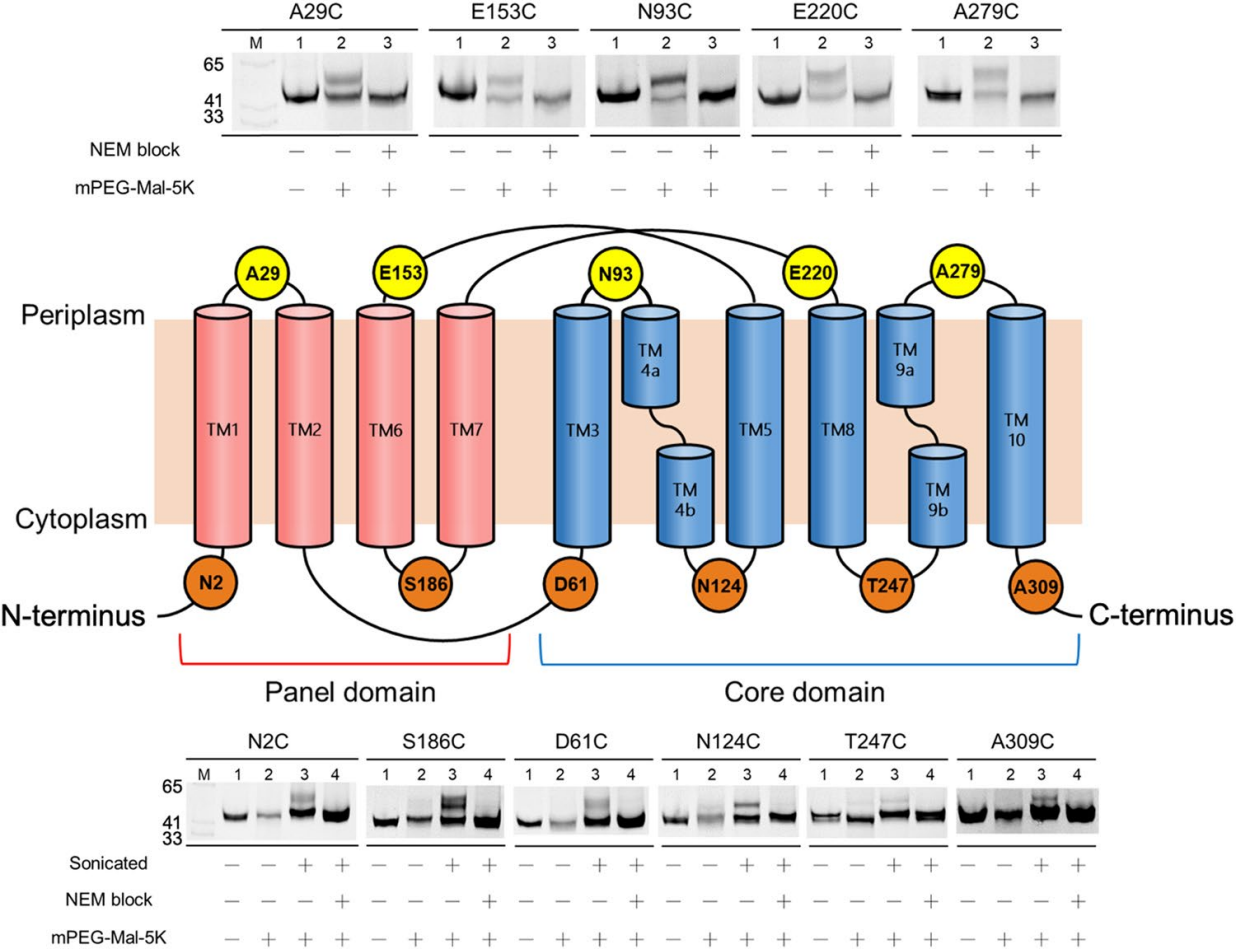
SDAF reveals a topological map of ASBT_NM_ in accordance with the crystal structure. Top panel: whole cell in-gel fluorescence images of the cysteine mutants with indicated treatments. For NEM (+) experiments, the samples were treated with NEM before mPEG-MAL-5K. Middle panel: the topology map of ASBT_NM_ determined by the crystal structure. Red and blue helices represent the components of panel and core domains, respectively. Residues with numbers represent the sites where cysteine residues were introduced by single point mutagenesis. A29, E153, N93, E220 and A279 are located in the extracellular loops; N2, S186, D61, N124, T247 and A309 are located in the intracellular loops. Lower panel: in-gel fluorescence images of the cysteine mutants with indicated treatments. Membrane permeabilization is achieved by sonication (+), where the *E*. *coli* cells were disrupted before the following treatments. See Methods for details.

To verify the effectiveness of SDAF in mapping the topology of membrane proteins, we introduced cysteine residues in the extracellular (A29C, N93C, E153C, E220C and A279C) and intracellular (N2C, D61C, N124C, S186C, T247C and A309C) loops of cfASBT_NM_ on the basis of the crystal structure (PDB ID 3ZUY). Because the C-terminally fused EGFP can emit fluorescence only when the target membrane protein is folded properly, the fluorescent signal of engineered membrane proteins can be used as a determinant of folded proteins^21,22^. Since EGFP fluorescence emission of all cysteine mutants tested here were comparable to that of the wild-type fusion protein (Supplementary Fig. S5a), it can be concluded that all mutants were properly folded. Additionally, all the single cysteine mutants showed comparable taurocholate uptake activity (60~80%) against to WT ASBT_NM_ (Fig. S8a). The substituted cysteine residues at the extracellular side showed clear band shifts after mPEG-MAL-5K modification using intact cells (Fig. 3, top panel, lanes 2 and Supplementary Fig. S6). Furthermore, PEGylation was inhibited if the cysteines were initially blocked by NEM, indicating that the band shifts were cysteine-specific (Fig. 3, top panel, lanes 3 and Supplementary Fig. S6). The variation in the observed PEGylation levels was probably due to steric hindrance of lipid head group. In contrast, none of the cysteine replacements in the intracellular loops were PEGylated in whole cells, again demonstrating mPEG-MAL-5K is impermeable to the inner membrane (Fig. 3, lower panel, lanes 2 and Supplementary Fig. S6). However, all of these mutants revealed a significant band shift after disrupting the cell membrane by sonication, suggesting membrane permeabilization allows mPEG-MAL-5K to gain access to the internal cysteines (Fig. 3, lower panel, lanes 3 and Supplementary Fig. S6). In summary, SDAF genuinely identified the location of cysteine replacements in either extra- or intracellular loops and generated a topological map in perfect agreement with the crystal structure.

### PEGylation profiles of ASBT_NM_ cysteine mutants reveal the solvent accessibility of the substrate permeation pathway

**Figure 4:**
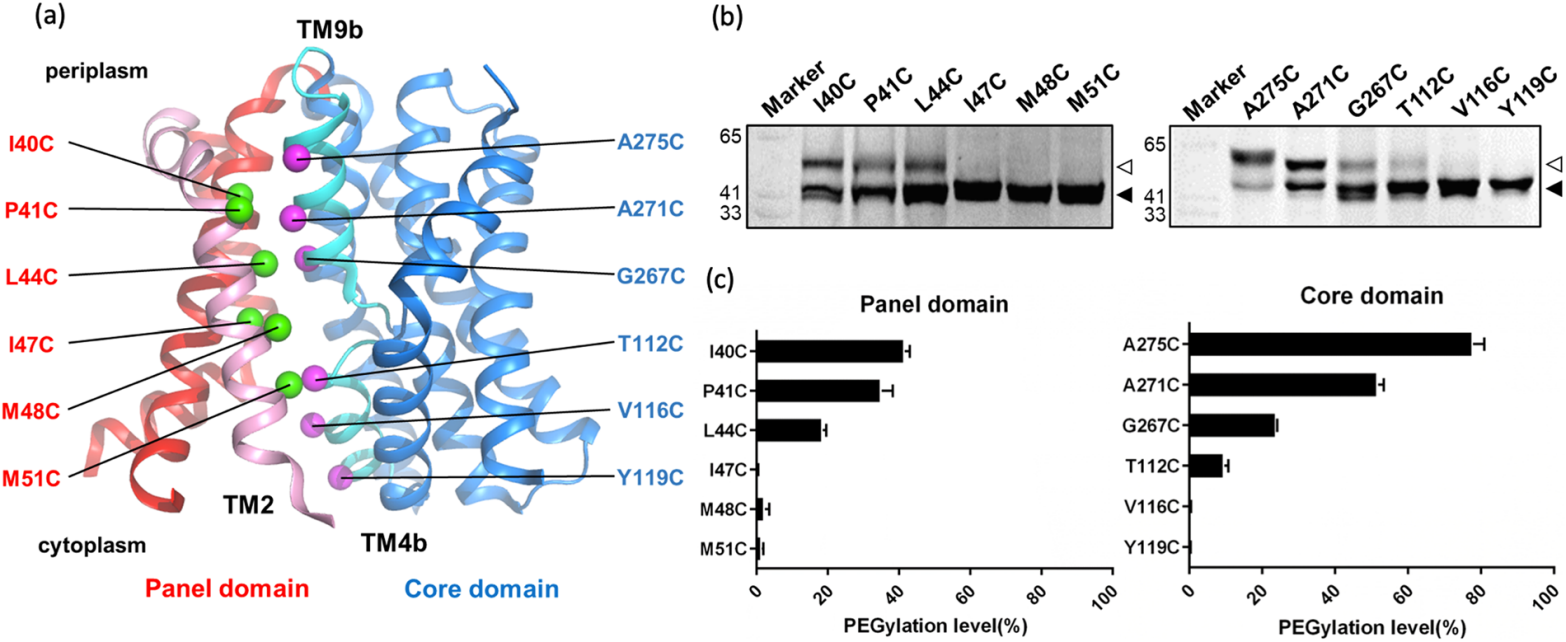
SDAF profiles reveal the solvent accessibility of residues lining the substrate translocation pathway. (**a**) Cartoon representation of the crystal structure of ASBT_NM_ (PDB 3ZUX) viewed from the membrane plane. The helices consisting of panel and core domains are coloured red and blue, respectively. TM6 helix is omitted for clarity. C_β_ atoms of the residues (I40, L44, I47, M48 and M51 on TM2 helix; A275, A271 and G267 on TM9b helix; and T112, V116 and Y119 on TM4b helix) replaced with cysteine are shown as green and magenta spheres, respectively. TM2 helix (panel domain) is coloured pink. TM9b and TM4b helices (core domain) are coloured cyan. (**b**) In-gel fluorescence using the *E*. *coli* whole cell expressing the cysteine mutants after the treatment of mPEG-MAL-5K. (**c**) The PEGylation levels were calculated and plotted as SDAF profiles. Empty and filled triangles indicate the position of PEGylated and non-PEGylated protein bands, respectively. The error bars represent the standard deviation of PEGylation efficiencies calculated from three independent experiments.

As the molar ratio of protein and labelling reagent can be precisely controlled based on the fluorescence emission arising from the EGFP fusion, it is possible to perform a quantitative analysis of PEGylation efficiency in order to evaluate the solvent accessibility at each specific amino acid replacement position. The crystal structures of two bacterial bile acid transporter homologues, ASBT_NM_ and ASBT_Yf_, illustrate the location of the substrate permeation pathway which is situated between the panel (helices TM1, TM2, TM6 and TM7) and core domains (helices TM3-TM5 and TM8-TM10). Helix TM2 in the panel domain packs against the discontinuous helices TM9b and TM4b in the core domain, contributing to the interface regions of the two domains. We chose the amino acids on helices TM2 (I40, P41, L44, I47, M48 and M51), TM9b (A275, A271 and G267) and TM4b (T112, V116 and Y119) with the side chains pointing into the substrate permeation pathway for individual cysteine substitution (Fig. 4a). PEGylation treatment was performed using intact *E*. *coli* cells overexpressing the mutants (Supplementary Fig. S5b). The uptake activities of these mutants were assessed, revealing a significant variation (Supplementary Fig. S8b). These mutants, especially G267C, located on the putative substrate binding site on the ASBT_NM_ core domain midway across the membrane, revealed ~20% uptake activity, probably due to its important role in substrate binding. As shown in the in-gel fluorescence images, the band shift of the mutants (A275C and I40C) positioned on the extracellular side was the most prominent (Fig. 4b and Supplementary Fig. S7). PEGylation efficiency decreased as the position of the cysteine replacement proceeds toward the intracellular side. The PEGylation efficiency at each position was calculated and plotted as a PEGylation profile (Fig. 4c). As the replacements positioned below I47C (TM2) on the panel domain and V116C (TM4b) on the core domain showed extremely low PEGylation efficiencies, these positions (I47C, M48C, M51C, V116C and Y119C) can be inferred as not being solvent accessible from the extracellular side of intact cells. Based on these results, one can conclude that the lower regions of TM2 and TM4b are packed tightly, consistent with the outward-facing crystal structure of ASBT_Yf_-E254A^19^. Furthermore, the data also corroborate the principle of alternating access mechanism of transporters: the substrate binding site in the core is exposed to either side of the membrane in an alternating fashion, but never simultaneously. In summary, the quantitative analysis delivering a PEGylation profile provides a useful method to evaluate the solvent accessibility of the substrate permeation pathway in native cell membrane environment.

## Discussion

Previously reported cysteine-scanning mutagenesis methods provided useful tools to obtain low-resolution structural information of membrane proteins, including topology maps, helix bundle packing and characterization of the substrate-binding site. However, the time-consuming and labour-intense protocols required by these methods make them less practical for quantification of the amount of target proteins and the engineered cysteine modifications. SDAF constitutes a substantial advancement of such methods and addresses the current shortcomings without the need for Western blotting or protein purification for visualization of target membrane proteins. Instead, this new methodology employs site-directed alkylation with mPEG-MAL-5K and can be performed using *E*. *coli* whole cells overexpressing the target protein fused with EGFP^21,23^. As a result, the PEGylated and non-PEGylated target membrane proteins can be distinctly detected by SDS-PAGE gel using in-gel fluorescence, rendering the EGFP fusion protein in its folded state and thus capable of emitting fluorescence during/after the electrophoresis. The assay itself can be done in ~3 hrs, therefore significantly improving the throughput of panels of cysteine mutants. Importantly, the molar ratio of target and reagent can be easily controlled by analysing the whole cell EGFP fluorescence counts. Therefore, the mPEG-MAL-5K labelling efficiencies of each cysteine mutant are pre-normalized and can be quantitatively compared. Carrying out such quantitative analysis in previously published methods has been proven very challenging, thus rendering quantitative analysis with those protocols inconsistent.

As mPEG-MAL-5K cannot permeate the inner membrane, the cysteine residues introduced in the intracellular loop showed no band shift using *E*. *coli* whole cells for PEGylation. Cell lysis using sonication resulted in gaining access of mPEG-MAL-5K to the intracellular cysteine residues. We did not use detergents to lyse cell membrane for two reasons: (1) some detergents may disrupt the native folding of membrane proteins, and (2) detergent micelles may introduce steric hindrance preventing the contact of the alkylation reagent with the cysteine sulfhydryl group. Potentially, inside-out vesicles (ISOVs) could be employed to allow access to the cysteine residues residing in the intracellular loops; however, the preparation of ISOVs requires further characterization to confirm the formation of uniformly oriented vesicles.

As folded EGFP has two endogenous cysteines, C48 and C70 at the reduced form, an accurate control of the concentration of the sulfhydryl reagent is required while performing SDAF experiments. We have shown that mPEG-MAL-5K is not permeable to the inner membrane of intact *E*. *coli* cells (Fig. 2c and Supplementary Fig. S3b). However, in the presence of extremely high concentration of mPEG-MAL-5K, such as the condition at 1:90,000 protein to mPEG-MAL-5K molar ratio, a band shift above the band of free EGFP is becoming visible (Supplementary Fig. S1b), suggesting excessive mPEG-MAL-5K may attach to the two endogenous cysteines in EGFP, although their accessibility to sulfhydryl reagents is limited in properly folded state^20^. While performing SDAF experiments using disrupted membranes, the concentration of mPEG-MAL-5K is added at much lower level because the membrane barrier is impaired and the cysteines on EGFP are more exposed to the sulfhydryl reagent. In this study, we used the protein to mPEG-MAL-5K ratios of 1:70,000 and 1:5,000 for whole cells and sonicator-disrupted membranes, respectively. To avoid the uncertainty in data interpretation, one has to perform extensive control experiments to determine the optimal molar ratio.

Although the use of C-terminally fused EGFP as the reporter has proven to be of great value in screening expression conditions and detergents for integral membrane proteins, it imposes a fundamental constraint: the target protein must have an intracellular C-terminus (C_in_ topology), otherwise the fused EGFP is not fluorescent. Extracellularly located EGFP has lost its ability to fluoresce due to being secreted in the unfolded state via the Sec system. Arriving in the oxidizing environment of the periplasm as an unfolded polypeptide chain, the two cysteine residues, C48 and C70, form intermolecular disulphide bonds which prevents folding of the β-barrel and formation of the fluorophore. A prediction of topologies of membrane proteins from 29 whole genome sequences demonstrated that 35% of multi-spanning membrane proteins have C_out_ topology^24^ which, at present, are not amenable to the technique introduced here. To overcome this problem, a method could be employed that allows to convert the topology of membrane proteins from C_out_ to C_in_ by fusing glycophorin A (GpA), a single membrane-spanning protein, on the C-terminus of the target protein^25^. The authors of that study also demonstrated that, using GpA as the fusion, the C-terminus is redirected into the cytoplasm, allowing the downstream GFP to become fluorescent, and the functionality was not impaired by the large fusion tag. Alternatively, an EGFP variant called ‘superfolder GFP’ (sfGFP) could be used instead of EGFP; sfGFP revealed faster folding kinetics and remained fluorescent in oxidizing environments^26^. It is also possible to utilize a cysteine-free GFP (cfGFP) variant that has been shown to possess comparable fluorescent brightness^27^. In future studies, replacing the EGFP gene with sfGFP or cfGFP in the pWaldo vector may enable membrane proteins with C_out_ topology to be fluorescent for SDAF assays.

In the present study, we introduced SDAF as a convenient tool for mapping the topology of membrane proteins and showed that the PEGylation levels of cysteine mutants can be easily quantified using SDAF, and the levels are indicative of the solvent accessibility of the given location in the protein. Therefore, SDAF can be applied to study conformational changes of membrane proteins in native cell membranes. Kaback and coworkers have developed a series of alkylation methods using radioactive or fluorescent sulfhydryl-reactive reagents^16,28^ (isotope-labelled NEM and TMRM), and demonstrated that ligand binding increases alkylation reactivity of cysteine replacements on the periplasmic side of LacY; i.e., LacY shifts to the outward-facing conformation in the presence of ligand. We also showed that PEGylation profiles obtained using the SDAF methodology provides a sensitive means to portray the substrate permeation pathway and may be applied to study substrate-induced dynamics of transporters. This can be achieved by comparing the PEGylation profiles using intact cells as well as permeabilized membranes, in the presence and absence of ligands and coupled ions. The systematic analysis based on data obtained with the SDAF methodology thus facilitates an understanding of alternating access mechanism for secondary active transporters.

In summary, SDAF is a versatile method allowing an efficient topology determination of multi-spanning membrane proteins, with α-helical or β-barrel folds, in a native membrane environment. The experimental data can be used to verify the *in silico* topology prediction of membrane proteins without known atomic structure. Additionally, the PEGylation profiles generated by SDAF can also be utilized in the studies of conformational dynamics of either soluble and membrane proteins.

## Methods

### Plasmid construction

The expression plasmid pWaldo-ASBT_NM_-EGFP-His_8_ containing the DNA fragment encoding ASBT_NM_ was constructed as previously described^17^. The two native cysteine residues of ASBT_NM_, Cys107 and Cys108, were substituted with serine using QuickChange Lightning Site-Directed Mutagenesis kit (Agilent). The cysteine-free mutant protein, termed pWaldo-cfASBT_NM_-EGFP-His_8_ was used as template for single cysteine mutagenesis at positions A29, N93, E153, E220 and A279 residing in the extracellular loops, and N2, D61, N124, S186, T247 and A309 located in the intracellular loops. For evaluation of the solvent accessibility of the substrate permeation pathway constituted by helix TM2 of the panel domain and the discontinuous helices TM4b and TM9b of the core domain, we also systematically introduced individual cysteine residues at positions I40, P41, L44, I47, M48 and M51 (helix TM2, panel domain), T112, V116 and Y119 (helix TM4b, core domain), and N265, G267, A271 and A275 (helix TM9b, core domain), of which the side chains are facing the substrate permeation pathway (Fig. 4a). Each mutant was confirmed by DNA sequencing of the nucleotide sequence encoding ASBT_NM_.

### Expression of cfASBT_NM_-EGFP and mutants

*Escherichia coli* C43(DE3) transformed with pWaldo containing cfASBT_NM_ with one single point mutation was grown in 10 mL LB broth in the presence of 50 μg/mL kanamycin at 37 °C. The overnight cultures were diluted 50-fold in 10 mL LB broth and ASBT_NM_ mutants were induced with 0.4 mM IPTG when the OD_600_ reached 0.4, followed by overnight growth at 25 °C. 1 mL of overnight culture was subjected to centrifugation at 13,000 rpm using a benchtop centrifuge. The pellet was resuspended in 100 μL 1 × PBS and transferred to a 96-well microplate (Garnier) for fluorescence intensity measurement (λ_exc_ = 485 nm, λ_em_ = 512 nm) using a spectrofluorometer (TECAN). The fluorescence counts were converted to EGFP concentration using an in-house standard curve^29^.

### Labelling with mPEG-MAL-5K

Approximately 1.4 μg EGFP worth of culture was taken from the rest of the overnight culture for mPEG-MAL-5K labelling. As the molecular weights of EGFP and cfASBT_NM_ in the pWaldo-ASBT_NM_-EGFP-His_8_ construct are 27 kDa and 32 kDa, respectively, 1.4 μg of EGFP corresponded to 1.6 μg of ASBT_NM_ in the fusion construct. The aliquoted culture was subjected to centrifugation and washed two times using PEGylation buffer (20 mM HEPES pH 7.5, 150 mM NaCl, 10% (v/v) glycerol). The pellet was resuspended in 350 μL PEGylation buffer and methoxypolyethylene glycol maleimide 5,000 (mPEG-MAL-5K, Sigma-Aldrich) was added at a final concentration of 10 mM. Hence, the molar ratio of ASBT_NM_ to mPEG-MAL-5K was approximately 1:70,000. The mixture was incubated at 27 °C for 1 h in the dark. 20 mM β-ME was added to terminate the reaction, followed by centrifugation at 13,000 for 1 min. For control experiments, free cysteine residues were blocked by adding NEM at a molar ratio of 1:1 before mPEG-MAL-5K treatment.

### Disruption of cell membranes

For the mutants carrying engineered cysteine residues in the intracellular loops of ASBT_NM_ (N2C, D61c, N124C, S186C, T247C and A309C), the *E*. *coli* cell membrane was permeabilized using sonication. 60 mL of IPTG-induced culture of each ASBT_NM_ mutant was grown at 25 °C overnight and harvested by centrifugation as described above. The cell pellets were resuspeneded in 5 mL sonication buffer (50 mM Tris-HCl pH 8.0, 200 mM NaCl, 15 mM EDTA and 100 μM PMSF). The cell membranes were disrupted on ice using a probe sonicator (MISONIN XL-2020) at a power amplitude of 2.5 for 4 cycles of 45 s bursts within 60 s intervals. The remaining intact cells were removed by low speed centrifugation at 6,000 rpm for 10 min using a benchtop centrifuge. The supernatant was collected and the crude membranes were further collected using a benchtop ultracentrifuge at 43,000 rpm for 1 hr. The membrane pellet was resuspended in 5 mL PEGylation buffer using a Dounce glass homogenizer. The fluorescence count of the crude membranes was measured as mentioned above and the equivalent of 1.6 μg ASBT_NM_ of crude membranes was aliquoted. mPEG-MAL-5K was added to the crude membranes at a molar ratio of approximately 1:5,000 (ASBT_NM_:mPEG-MAL-5K). As a control experiment, free cysteine residues were blocked by adding NEM at a molar ration of 1:1 before mPEG-MAL-5K treatment.

### Imaging using in-gel fluorescence

In-gel fluorescence using whole cells was performed as described previously^23^. Briefly, the cell pellet was resuspended in 10 μL 1 × PBS and 10 μL 2× sample loading buffer (200 mM Tris-HCl pH 8.8, 20% (v/v) glycerol, 5 mM EDTA pH 8.8, 0.02% Bromophenol Blue, and 4% SDS). 0.3 μL Benzonase Nuclease (Sigma) was added to digest the exposed chromosomal DNA. The mixture was subjected to centrifugation at 15,000 rpm, the supernatant was loaded onto a 4–20% Tris-Glycine SDS-PAGE gel and electrophoresis was performed on ice. For in-gel fluorescence of disrupted cell membranes, 10 μL of PEGylated crude membranes were mixed with 10 μL sample buffer. The mixture was subjected to SDS-PAGE electrophoresis performed on ice. To detect the fluorescent band of cfASBT_NM_-EGFP fusion proteins, the SDS-PAGE gel was analysed densitometrically using the Gel Doc^TM^ EZ imaging system (Bio-Rad) and a blue tray. Densitometric analysis of the fluorescent bands was carried out using the software Image Lab (Bio-Rad). The labelling efficiency of a particular cysteine residue was calculated by dividing the fluorescence density of the shifted band by the total density of the shifted and non-shifted bands of ASBT_NM_-EGFP,

1 $${\rm{PEGylation}}\,{\rm{efficiency}}\,( \% )=\frac{{\rm{density}}\,{\rm{of}}\,{\rm{shifted}}\,{\rm{band}}}{{\rm{density}}\,{\rm{of}}\,{\rm{shifted}}\,{\rm{band}}+{\rm{density}}\,{\rm{of}}\,{\rm{non}} \mbox{-} {\rm{shifted}}\,{\rm{band}}} \% $$

### Substrate uptake activity assay

Substrate uptake activity of ASBT_NM_ mutants was conducted in *E*. *coli* C43(DE3) whole cells according to the previously published method^17^. Briefly, 1.5 mL LB culture of *E*. *coli* cells overexpressing the WT ASBT_NM_-EGFP and cysteine mutants were harvested and the amount of target protein was normalized based on the EGFP fluorescence counts. The cell pellets were resuspended in 150 μl uptake buffer consisting of 100 mM NaCl, 1 mM CaCl_2_, 1 mM MgCl_2_, 10 mM Tris-HCl pH:7.5. Cells were incubated at 37 °C with uptake buffer containing 4 μM taurocholate supplemented with 0.17 μM [^3^H(G)]-taurocholate (15.4 Ci mmol-1; Perkin Elmer) for 10 mins. Substrate uptake was terminated by adding 150 μL ice-cold stop buffer containing 100 mM NaCl, 1 mM CaCl_2_, 1 mM MgCl_2_, 10 mM Tris-HCl pH:7.5, 1 mM Taurocholic Acid immediately followed by vacuum filtration (QIAvac 24 Plus Vacuum Manifold, Qiagen) and four cycles of wash using 5 mL uptake buffer. The radioactivity of the internalized [^3^H(G)]-taurocholate was measured using scintillation counting. Each uptake experiment was performed in triplicate. The basal uptake was measured in triplicate by using C43(DE3) *E*. *coli* transformed with pWaldo-EGFP. Specific uptake was obtained by the subtraction of basal uptake from total uptake.

## Supplementary information

Supplementary information
