# Supplementary material for: Site-Directed Alkylation Detected by In-Gel Fluorescence (SDAF) to Determine the Topology Map and Probe the Solvent Accessibility of Membrane Proteins
Source: Sci Rep. 2019 Sep 11;9:13171. doi: 10.1038/s41598-019-49292-w (PMC6739316; doi:10.1038/s41598-019-49292-w)
Supplement: Supplementary file 1 — Supplementary information [file 41598_2019_49292_MOESM1_ESM.docx]

**Site-Directed Alkylation Detected by In-Gel Fluorescence (SDAF) to Determine the Topology Map and Probe the Solvent Accessibility of Membrane Proteins**

Yu-Hung Lin^1^, Sung-Yao Lin^1^, Guan-Syun Li^1^, Shao-En Weng^1^, Shu-Ling Tzeng^2^, Yu-Hsuan Hsiao^1^, Nien-Jen Hu^1,3*^

^1^ Graduate Institute of Biochemistry, National Chung Hsing University, 145 Xinda Rd., South Dist., Taichung City 402, Taiwan, R.O.C.

^2^ Institute of Medicine, Chung Shan Medical University, No.110, Sec. 1, Jianguo N. Rd., Taichung City 40201, Taiwan, R.O.C.

^3^ Rong Hsing Research Center for Translational Medicine, National Chung Hsing University, 145 Xinda Rd., South Dist., Taichung City 402, Taiwan, R.O.C.

*Corresponding author

email: njhu@nchu.edu.tw

**Supplementary information**

**
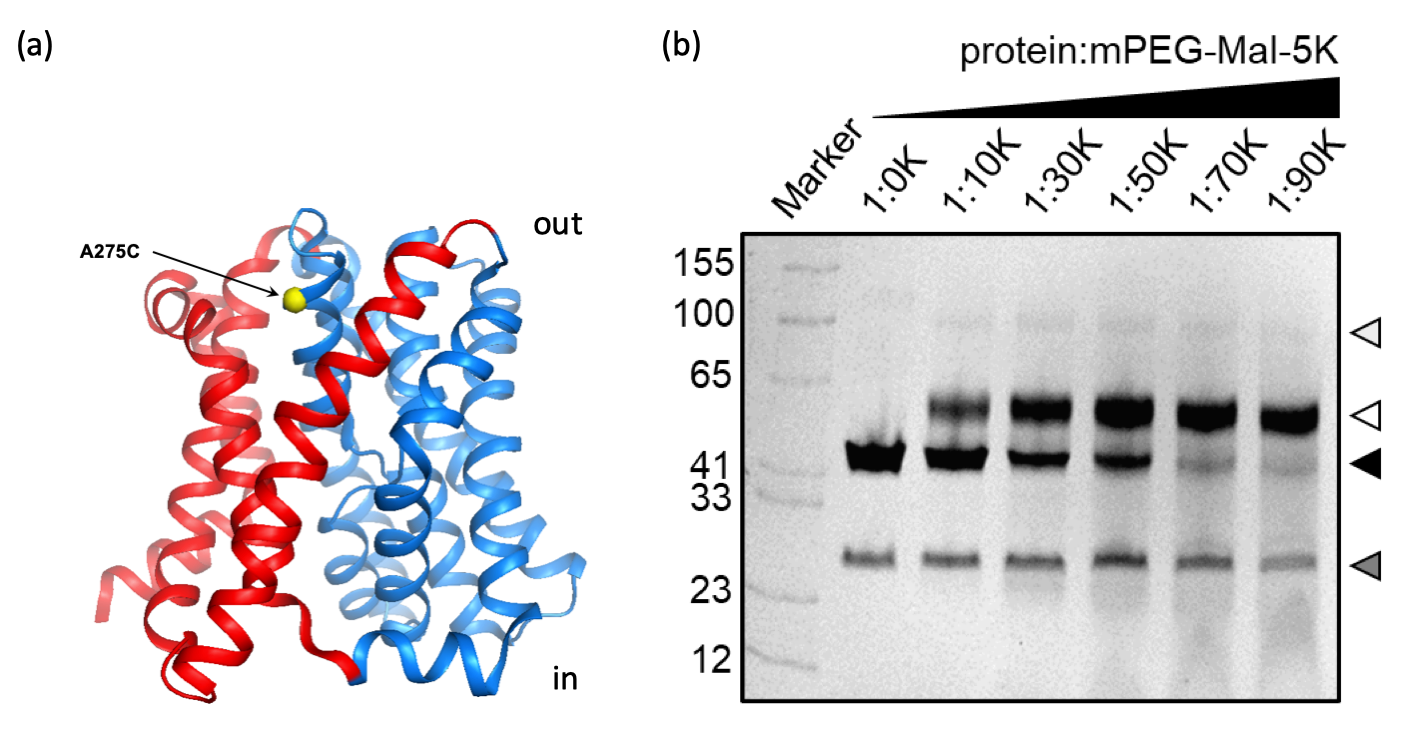
**

**Supplementary Fig. S1**

PEGylation of cfASBT_NM_ A275C-EGFP using whole-cell in-gel fluorescence. (a) The crystal structure of ASBT_NM_ with the panel and core domains colored in red and blue, respectively. The Cα of A275 is shown as yellow sphere. (b) The complete in-gel fluorescence image of PEGylated cfASBT_NM_ A275C-EGFP with indicated protein to mPEG-MAL-5K molar ratios using *E. coli* whole cells as shown in Fig. 2a. Empty and filled triangles indicate the position of PEGylated and non-PEGylated protein bands, respectively. Dark grey triangle: the free EGFP produced by non-specific proteolysis during cell lysis. This is due to non-specific protease activity after cell lysis. Light grey triangle: ASBT_NM_-EGFP at higher oligomeric states.


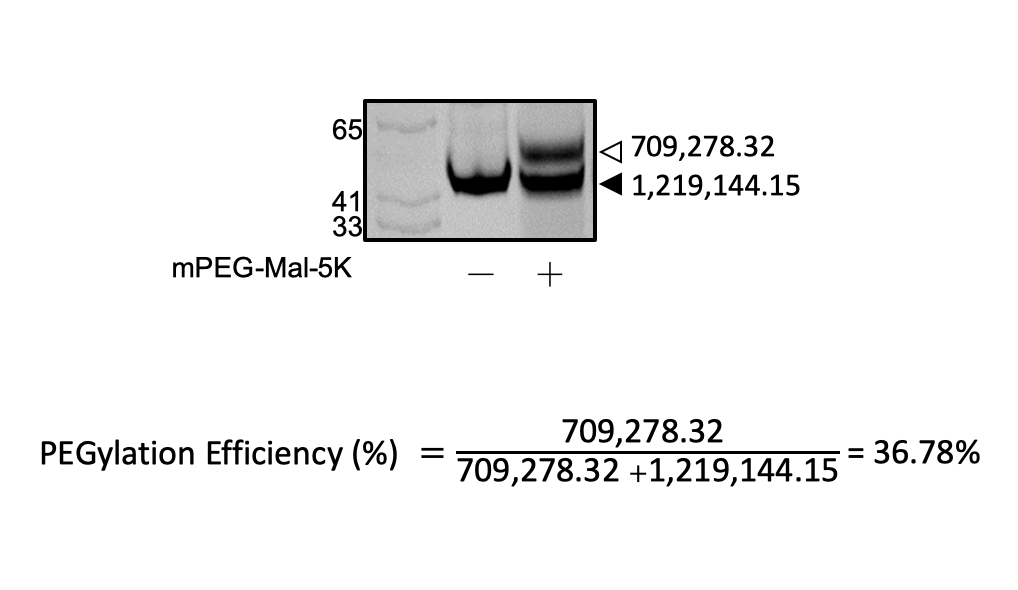


**Supplementary Fig. S2**

An example of calculating PEGylation Efficiency of cfASBT_NM_ (A29C)-EGFP using SDAF. In the absence of mPEG-MAL-5K (-), the single band indicated by the black triangle represents the nonPEGylated cfASBT_NM_ (A29C)-EGFP. In the presence of mPEG-MAL-5K (+), a band shift revealing an increase of 5K in mw (indicated by the white triangle) appears, representing the PEGylated cfASBT_NM_ (A29C)-EGFP. Densitometric analysis is performed as described in Methods and the densities are shown. The PEGylation Efficiency is calculated from Equation (1), revealing an efficiency of 36.68%.


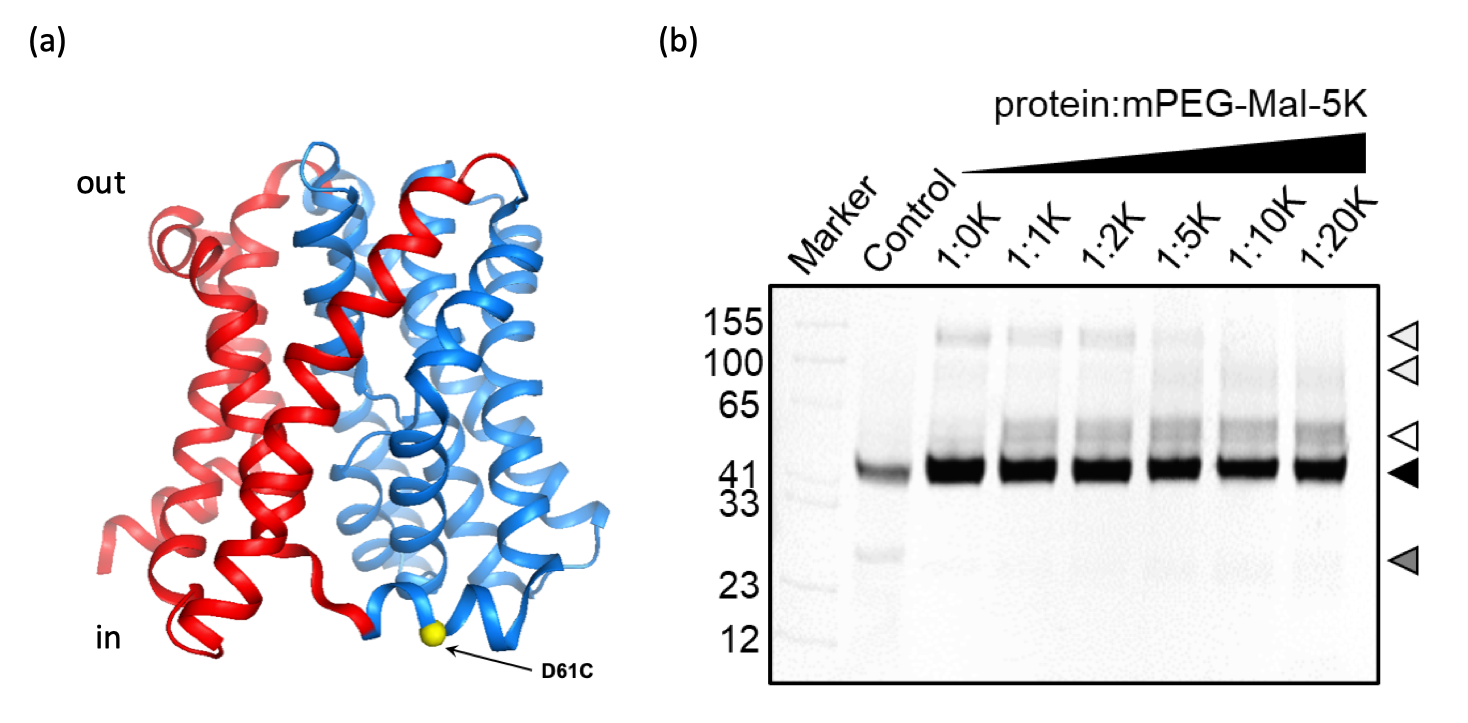


**Supplementary Fig. S3**

PEGylation of cfASBT_NM_ D61C-EGFP using permeabilized membranes for in-gel fluorescence. (a) The crystal structure of ASBT_NM_ with the panel and core domains colored in red and blue, respectively. The Cα of D61 is shown as yellow sphere. (b) The complete in-gel fluorescence image of PEGylated cfASBT_NM_ D61C-EGFP with indicated protein to mPEG-MAL-5K molar ratios using permeabilized membranes as shown in Fig. 2c. Empty and filled triangles indicate the position of PEGylated and unPEGylated protein bands, respectively. Dark grey triangle: the free EGFP produced by non-specific proteolysis during cell lysis. The amount of free EGFP is lower compared to that in Supplementary Fig. S1 is because the free EGFP is removed from the membrane pellet after ultracentrifugation. Light grey triangle: ASBT_NM_-EGFP at higher oligomeric states.

**
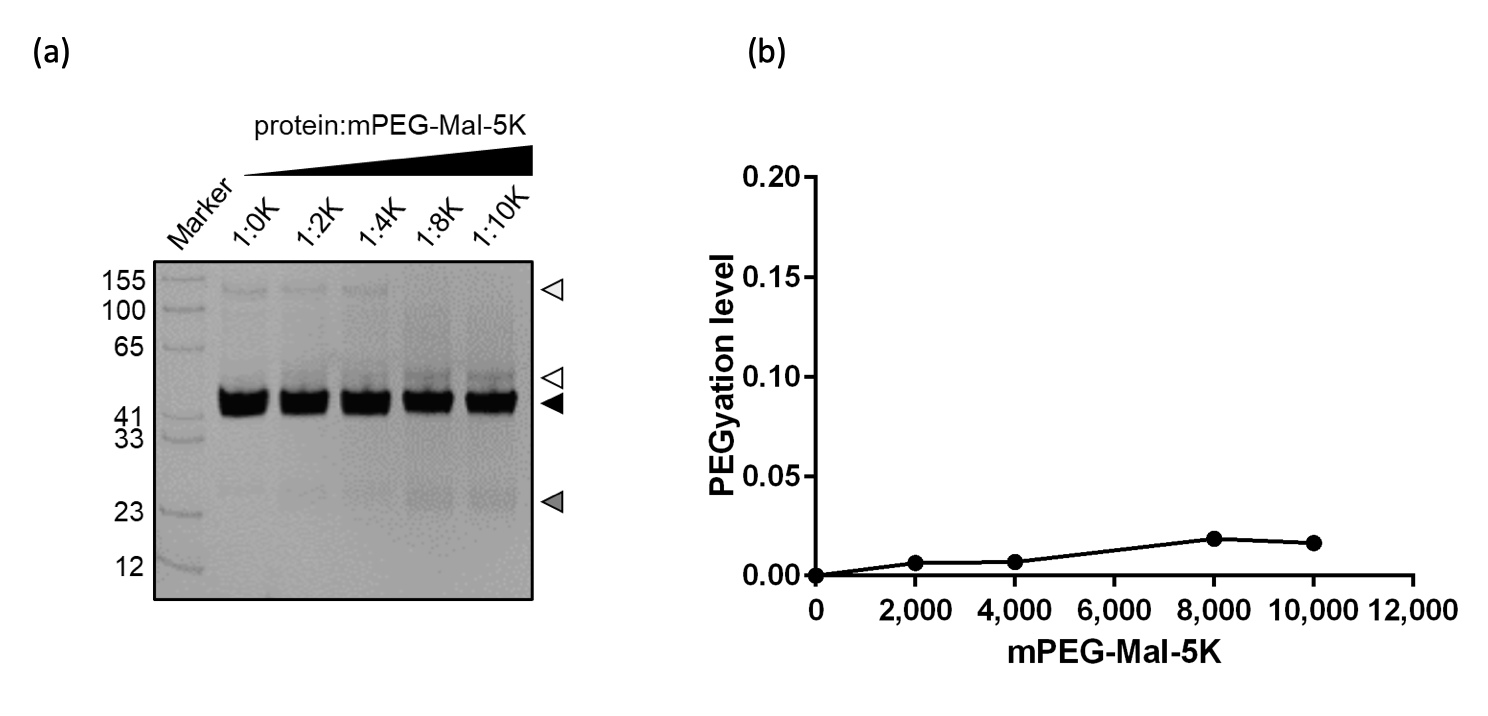
**

**Supplementary Fig. S4**

A control experiment demonstrating that the PEGylation of two native cysteine residues in EGFP is negligible. (a) The in-gel fluorescence shows the PEGylation levels of permeabilized membranes containing cfASBT_NM_-EGFP incubated with indicated protein to mPEG-MAL-5K molar ratios. (b) The PEGylation levels plotted against indicated protein to mPEG-MAL-5K molar ratios. Empty and filled triangles indicate the position of PEGylated and non-PEGylated protein bands, respectively. Dark grey triangles: the free EGFP produced by non-specific proteolysis during cell lysis. Light grey triangles: ASBT_NM_-EGFP at higher oligomeric states.

**
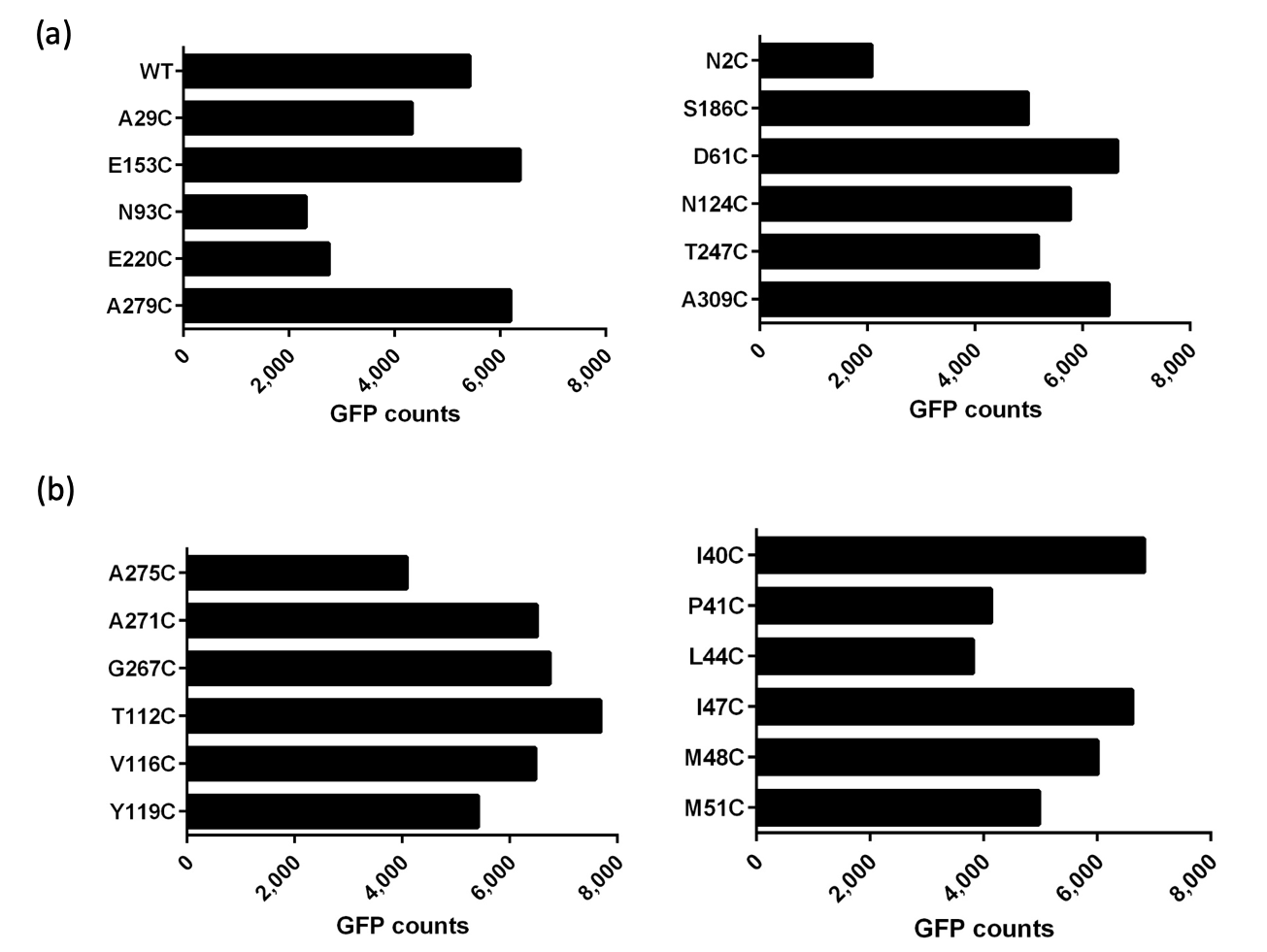
**

**Supplementary Fig. S5**

Whole-cell fluorescence counts of *E. coli* cells overexpressing WT ASBT_NM_-EGFP and cysteine mutants. The counts were obtained as described in Methods. (a) The histogram of fluorescence counts of the ASBT_NM_ cysteine mutants generated for the topological mapping experiment using SDAF as shown in Fig. 3. The cysteine residues introduced in the extra- and intracellular loops are shown in the left and right panels, respectively. (b) The histogram of fluorescence counts of the ASBT_NM_-EGFP cysteine mutants generated for the solvent accessibility assay using SDAF as shown in Fig. 4. The cysteine residues introduced in the panel and core domains are shown in left and right panels, respectively.

**
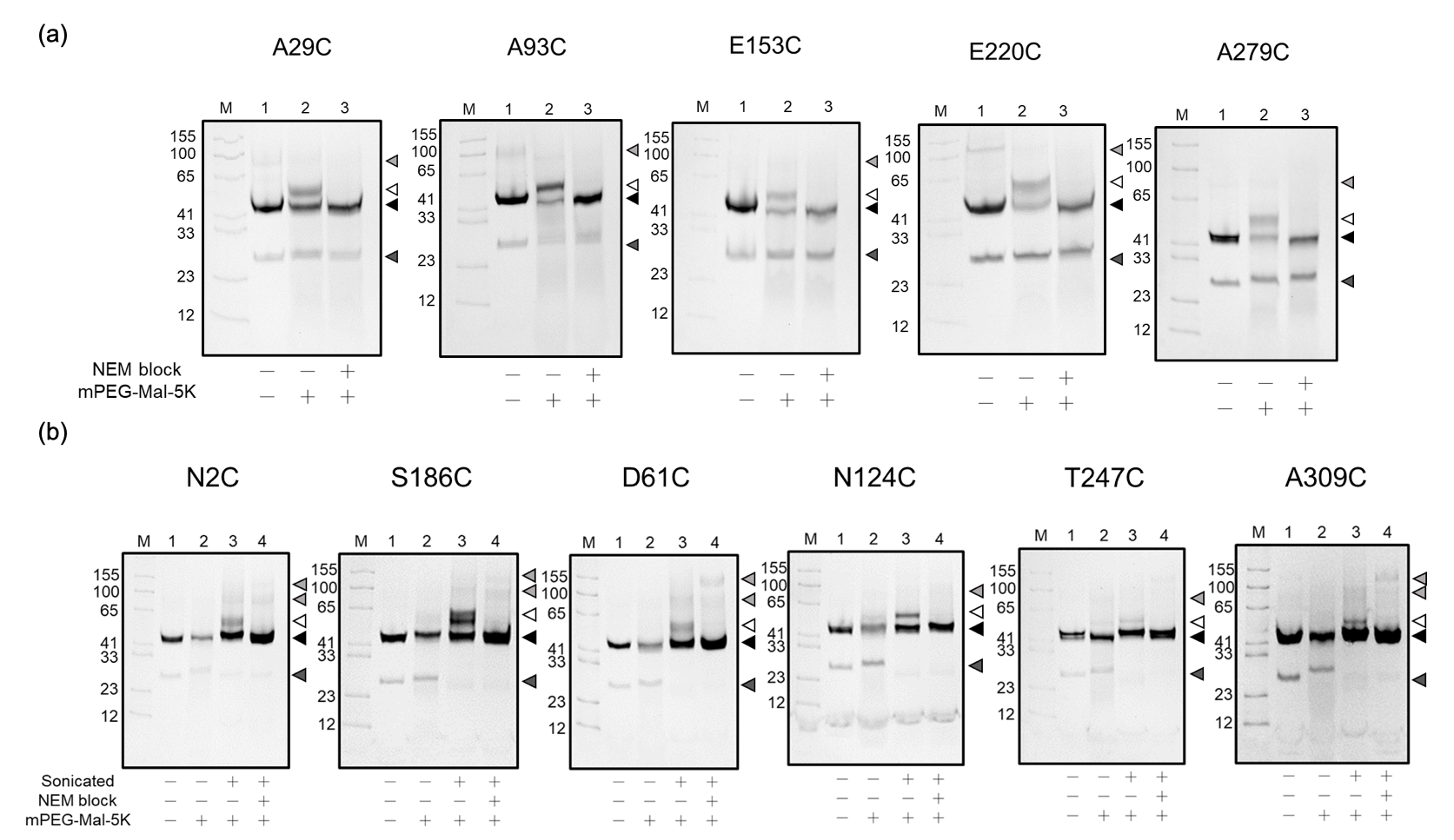
**

**Supplementary Fig. S6**

The complete in-gel fluorescence images of the ASBT_NM_ cysteine mutants for topology mapping as shown in Fig. 3. The in-gel fluorescence images of the ASBT_NM_ mutants with the cysteine residue introduced on the extracellular (a) and intracellular loops (b) with the indicated treatments. Empty and filled triangles indicate the position of PEGylated and non-PEGylated protein bands, respectively. Dark grey triangles: the free EGFP produced by non-specific proteolysis during cell lysis. Light grey triangles: ASBT_NM_-EGFP at higher oligomeric states.

**
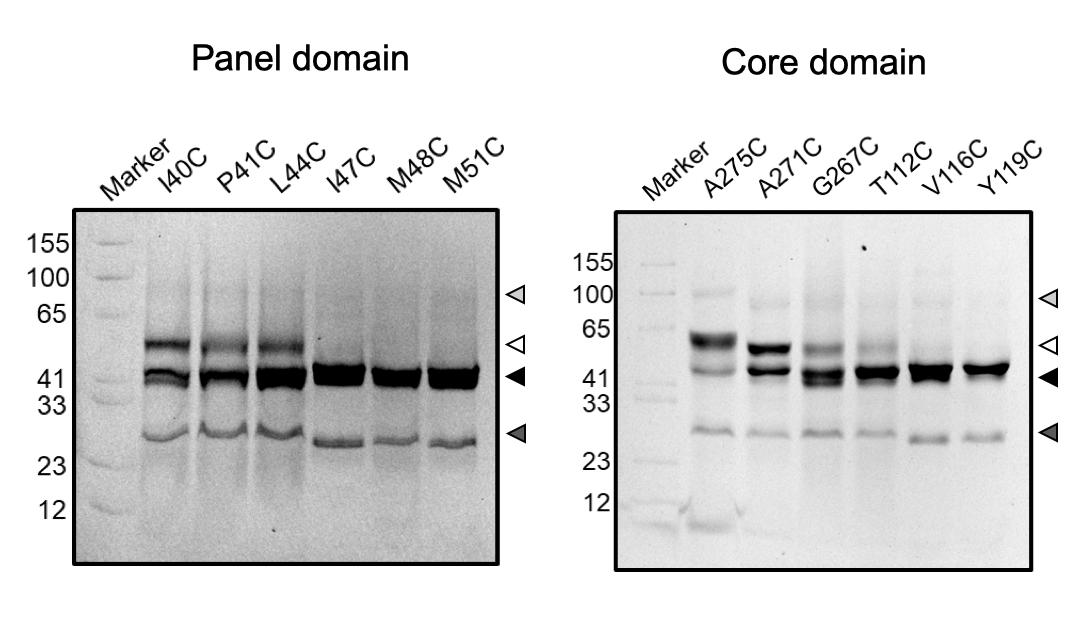
**

**Supplementary Fig. S7**

The complete in-gel fluorescence images of the ASBT_NM_ cysteine mutants for solvent accessibility probing as shown in Fig. 4. Empty and filled triangles indicate the position of PEGylated and non-PEGylated protein bands, respectively. Dark grey triangles: the free EGFP produced by non-specific proteolysis during cell lysis. Light grey triangles: ASBT_NM_-EGFP at higher oligomeric states.

**
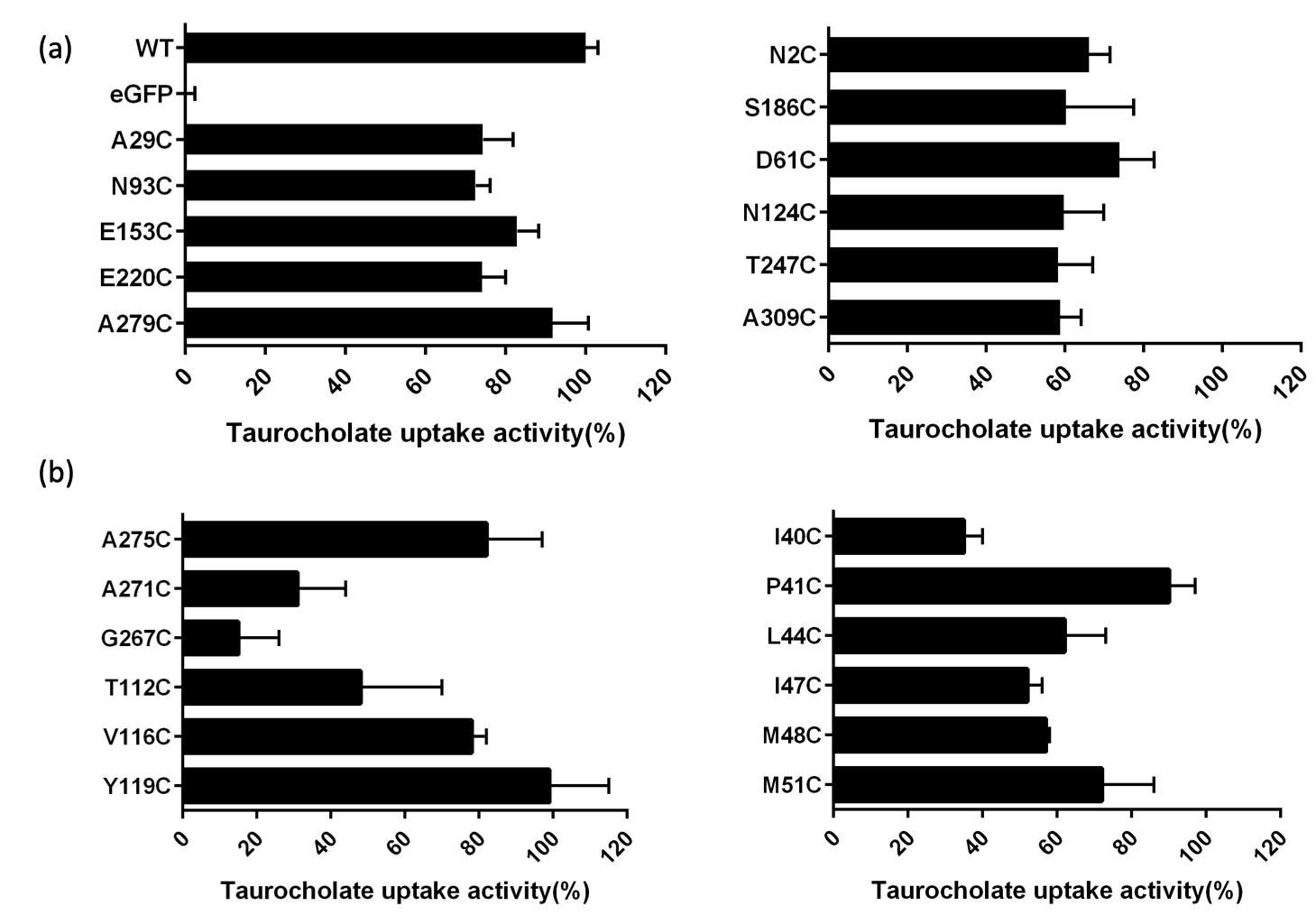
**

**Supplementary Fig. S8**

Taurocholate uptake activities of WT ASBT_NM_-EGFP and cysteine mutants. The substrate uptake assay is described in Methods. The basal uptake activity was measured using *E. coli* C43(DE3) transformed with pWaldo-EGFP vector, and was wet to 0% activity. The specific uptake of WT ASBT_NM_-EGFP was calculated by subtraction of basal uptake from total uptake activity, and was set to 100% activity. (a) The uptake activities of the ASBT_NM_ cysteine mutants generated for the topological mapping experiment using SDAF as shown in Fig. 3 and Fig. S6. The cysteine residues introduced in the extra- and intracellular loops are shown in the left and right panels, respectively. (b) The uptake activities of the ASBT_NM_-EGFP cysteine mutants generated for the solvent accessibility assay using SDAF as shown in Fig. 4 and Fig. S7. The cysteine residues introduced in the panel and core domains are shown in left and right panels, respectively. Error bars represent the standard deviation of uptake activities calculated from three independent experiments.
